# Using electronically delivered therapy and brain imaging to understand OCD pathology: A pilot feasibility study

**DOI:** 10.3389/fpsyt.2023.1050530

**Published:** 2023-03-09

**Authors:** Callum Stephenson, Niloufar Malakouti, Joseph Y. Nashed, Tim Salomons, Douglas J. Cook, Roumen Milev, Nazanin Alavi

**Affiliations:** ^1^Centre for Neuroscience Studies, Faculty of Health Sciences, Queen’s University, Kingston, ON, Canada; ^2^Department of Psychiatry, Faculty of Health Sciences, Queen’s University, Kingston, ON, Canada; ^3^Department of Medicine, School of Medicine, Queen’s University, Kingston, ON, Canada; ^4^Department of Psychology, Faculty of Arts and Sciences, Queen’s University, Kingston, ON, Canada; ^5^Neurosurgery Division, Department of Surgery, School of Medicine, Queen’s University, Kingston, ON, Canada; ^6^Department of Psychiatry, Providence Care Hospital, Kingston, ON, Canada

**Keywords:** obsessive–compulsive disorder, psychotherapy, neuroimaging, cognitive behavioral therapy, exposure response prevention, neural pathology

## Abstract

**Background:**

Obsessive–compulsive disorder (OCD) is a debilitating mental health disorder with current psychotherapeutic treatments, while somewhat effective, yielding low accessibility and scalability. A lack of knowledge regarding the neural pathology of OCD may be hindering the development of innovative treatments. Previous research has observed baseline brain activation patterns in OCD patients, elucidating some understanding of the implications. However, by using neuroimaging to observe the effects of treatment on brain activation, a more complete picture of OCD can be drawn. Currently, the gold standard treatment is cognitive behavioral therapy (CBT). However, CBT is often inaccessible, time-consuming, and costly. Fortunately, it can be effectively delivered electronically (e-CBT).

**Objectives:**

This pilot study implemented an e-CBT program for OCD and observed its effects on cortical activation levels during a symptom provocation task. It was hypothesized that abnormal activations could be attenuated following treatment.

**Methods:**

OCD patients completed a 16-week e-CBT program administered through an online platform, mirroring in-person content. Treatment efficacy was evaluated using behavioral questionnaires and neuroimaging. Activation levels were assessed at the resting state and during the symptom provocation task.

**Results:**

In this pilot, seven participants completed the program, with significant improvements (*p* < 0.05) observed between baseline and post-treatment for symptom severity and levels of functioning. No statistically significant (*p* = 0.07) improvement was observed in the quality of life. Participants had mostly positive qualitative feedback, citing accessibility benefits, comprehensive formatting, and relatable content. No significant changes in cortical activation were observed between baseline and post-treatment.

**Conclusion:**

This project sheds light on the application of e-CBT as a tool to evaluate the effects of treatment on cortical activation, setting the stage for a larger-scale study. The program showed great promise in feasibility and effectiveness. While there were no significant findings regarding changes in cortical activation, the trends were in agreeance with previous literature, suggesting future work could provide insight into whether e-CBT offers comparable cortical effects to in-person psychotherapy. Applying a greater knowledge of the neural mechanisms of action in OCD can help develop novel treatment plans in the future.

## 1. Introduction

Obsessive–compulsive disorder (OCD) is a debilitating disorder with a lifetime prevalence globally of 3% (1). Moreover, only half of the patients achieve remission (2). A lack of understanding regarding the pathology of OCD could be contributing to the lack of treatment effectiveness. By understanding the pathology, more targeted treatments could be developed in the future, leading to innovative solutions with better treatment outcomes. One way to accomplish this could be to observe the effects of treatment using neuroimaging.

Currently, cognitive behavioral therapy (CBT) with exposure and response prevention (ERP) is the gold standard treatment for OCD (3–5). The structure of CBT for OCD mirrors many aspects of its counterpart for depression and anxiety, with the main differentiator being the incorporation of ERP. Although CBT is a gold standard, its in-person delivery comes with high costs, and a large time commitment from healthcare providers which reduces scalability, long wait lists, and geographic and temporal inaccessibility concerns for patients. Treatment is costly, time-consuming, and often inaccessible. Fortunately, due to the structured nature of CBT, it can be effectively delivered electronically, through the internet [e-CBT; (6–11)].

The exact pathology of OCD is currently unknown. However, one way to better understand neural pathology is to use functional MRI (fMRI) to assess brain activation levels in OCD patients. During fMRI, changes in blood flow to different cortical regions are measured while a subject performs a specific task. These tasks can include an image viewing scan (i.e., viewing a neutral image on a screen), a cognitive task (i.e., Stroop task), or a symptom provocation task (i.e., viewing images related to the patient’s anxieties). In patients with OCD, these tasks can help outline brain function during resting state, decision-making, or anxiety processing. The changes in cortical activation are measured as blood-oxygen-level-dependent (BOLD) changes (12). These BOLD changes can then be mapped to an expected hemodynamic response function (HRF), helping to identify cortical regions activated during different portions of the task (i.e., resting vs. symptom provocation) and time points (i.e., baseline vs. post-treatment).

Previous fMRI research on OCD patients has identified several cortical regions and circuits with abnormal activations (13). A review by Shephard et al. (13) compared baseline scans of healthy controls (HCs) and OCD patients and found that the fronto-limbic circuit (responsible for emotional response), which includes the orbitofrontal cortex, frontal gyrus, anterior cingulate cortex, and amygdala was hyperactive. This could contribute to the increased response to negative emotions commonly seen in OCD patients. Next, the sensorimotor circuitry (responsible for motor behavior and sensory integration), which includes the thalamus, putamen, precentral gyrus, and insula was found to be hyperactive. This could contribute to physical compulsions and abnormal sensitivity to sensory stimuli. The ventral cognitive circuit (responsible for behavioral control), which includes the thalamus, ventral caudate, inferior frontal gyrus, and ventrolateral prefrontal cortex was found to be hypoactive. This could help explain the inability to control compulsive behaviors seen in OCD patients. Moreover, the ventral affective circuit (responsible for reward processing), which includes the thalamus, nucleus accumbens, and orbitofrontal cortex, was also found to be hypoactive compared to HCs. This could present in OCD patients by using compulsions to avoid anxieties and increased fear of punishment. Finally, the dorsal cognitive circuit (responsible for executive function), which includes the thalamus, dorsal caudate, and the dorsolateral and dorsomedial prefrontal cortex, was found to be hypoactive. This could contribute to the maladaptive executive functioning commonly seen in OCD (13). Knowing which regions are abnormally activating, and what the roles and responsibilities of these regions are can help us develop a deeper understanding of OCD. Moreover, applying this knowledge to treatment development through psychotherapeutics targeting the behaviors connected to these abnormally functioning neural circuits may result in improved neuroplasticity and ultimately, better treatment outcomes (14). While the aforementioned circuits have been mainly observed at baseline in OCD patients, determining if and how treatment affects these circuits can provide further insight into OCD pathology (15, 16).

There is some previous research evaluating the effects of in-person psychotherapeutics on brain activation in OCD patients. In general, in-person treatment resulted in decreased activation in the frontal (orbitofrontal cortex, prefrontal cortex, and anterior cingulate cortex), parietal (precuneus, parietal cortex, supramarginal gyrus), and temporal lobe (caudate nucleus, nucleus accumbens, insula, parahippocampal gyrus), cerebellum, and vermis (12, 17–23). However, to the author’s knowledge, there is no previous knowledge on whether virtually-delivered therapy.

Using fMRI to evaluate the effects of CBT and ERP on brain activation during neural anxiety processing could provide further insight into the cortical pathology of OCD. Functional neuroimaging is not required to discern whether treatment was successful but is required to determine the mechanism of action (24). By having a clearer understanding of the neural mechanisms of action, more informed development of future treatments can be made for a population that is in urgent need of innovative interventions (25, 26).

## 2. Materials and methods

A non-randomized pilot study design was employed with all participants receiving 16 weekly sessions of e-CBT. fMRI was conducted at baseline and post-treatment to evaluate activation level changes. Clinically validated symptomology questionnaires were used to evaluate treatment efficacy. Additionally, qualitative interviews were conducted to gather personal demographic information as well as information regarding participant experience while using the online psychotherapy platform. The pilot study was registered on the ClinicalTrials.gov Protocol Registration and Results System (NCT04630197). Additionally, ethics approval was obtained from the Queen’s University Health Sciences and Affiliated Teaching Hospitals Research Ethics Board (HSREB; File Number 6031276).

### 2.1. Participants

Participants were recruited from family medicine and psychiatric clinics at Queen’s University and Kingston Health Sciences Centre sites (Hotel Dieu Hospital and Kingston General Hospital) in Kingston, Ontario, Canada. Additionally, local, and social media advertisements were utilized. Participants were enrolled in the study based on referrals from outpatient clinics, family doctors, as well as self-referrals. Those invited and interested in participating had the study protocol explained along with an evaluation by a psychiatrist on the research team through a secured video appointment. Participants were evaluated for a diagnosis of OCD based on the Diagnostic and Statistical Manual of Mental Disorders, Fifth Edition [DSM-5; (27)]. Once a diagnosis of OCD was confirmed and the participant was given written and verbal instructions on how to participate in the study, informed consent was obtained.

Inclusion criteria included the following: over the age of 18 years at the start of the study, a diagnosis of OCD according to DSM-5 criteria, competence to consent to participate, ability to speak and read English, and consistent and reliable access to the internet. Exclusion criteria included the following: having any metal implants or additional factors deemed not safe for an MRI scan, active psychosis, acute mania, severe alcohol, or substance use disorder, and/or active suicidal or homicidal ideation. Additionally, if a participant was currently receiving another form of psychotherapy, they were excluded from the study. If a participant was on medication, they had to have been on that dosage for at least the past 6 weeks and the dosage had to remain unchanged for the duration of the study.

### 2.2. Therapy

Weekly sessions of e-CBT were conducted through the Online Psychotherapy Tool (OPTT; OPTT Inc.), a secure, online, cloud-based mental health care delivery platform. These online sessions consisted of approximately 30 slides and interactive therapist videos, with 16 modules in total (1 module per week). The content and format of these online sessions mirrored in-person CBT for OCD (Table 1) (28). The connection between thoughts, behaviors, emotions, physical reactions, and the environment was a focus of module content. Moreover, mindfulness, body scanning, self-care, goal setting, thinking errors, the 5-part model, and thought records were employed as techniques for participants. ERP was incorporated into the e-CBT program as this is the first-line route of treatment. Slides highlighted different topics each week and included general information, an overview of skills, and homework on that topic. The homework included in each session was submitted through OPTT and reviewed by therapists with personalized feedback provided within 3 days of submission. Weekly homework submission for feedback was mandatory before being eligible for the next session. After each completion of the e-CBT program, participants provided feedback through OPTT on their perception of how the treatment went, and any pros and/or cons of the online format that they found. OPTT can be accessed from a variety of devices (i.e., desktop computer, laptop, cellphone, tablet, etc.) and internet browsers.

**Table 1:** Content summary for the 16-week e-CBT with ERP program for OCD.

Session #	Session title
1	What is cognitive behavioral therapy?
2	Obsessions and compulsions and how to recognize them
3	Measuring our anxiety
4	Why you cannot escape your obsessions and compulsions
5	Common misinterpretations in OCD
6	Introduction to exposure and response prevention
7	Customizing a personalized treatment plan
8	Exploring the treatment plan
9	Pros and cons of CBT for OCD
10	Setting goals for working on your OCD
11	Strategies you can use
12	Exposure therapy
13	Situational exposure menu
14	Imaginal exposure practice
15	Response prevention
16	Review

### 2.3. Imaging

All neuroimaging occurred at the Queen’s University MRI Facility in Kingston, Ontario, Canada using a Siemens PRISMA Fit 3.0 Tesla whole-body MRI scanner with a 32-channel standard coil. Scans occurred at baseline (pre-treatment) and after week 16 (post-treatment). During scanning, participants were instructed to lay still on the scanning table on their backs, with their heads resting on a foam pad to reduce movement. Scanning appointments took approximately 1 h per session.

**Figure 1:**
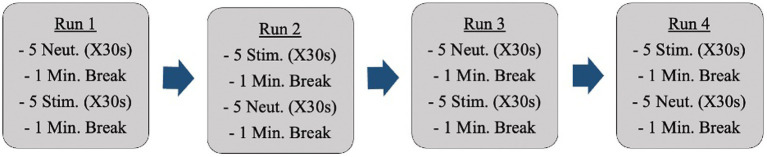
Breakdown of the sequence of IAPS images shown during the fMRI acquisition procedure.

Anatomical reference images were captured initially. Following this, fMRI scans occurred while participants were shown neutral images and anxiety-inducing images (i.e., dirty dishes if cleanliness was an anxiety-inducing concept for a specific participant). Participants were shown all images through a mirror back projection. Changes in activation during neural anxiety processing were analyzed. The images participants were shown came from a standardized photo bank provided by the International Affective Picture System [IAPS; (29)]. Each set of pictures was individually tailored to each participant’s obsessions and compulsions. These images were selected ahead of time by the primary investigator in collaboration with a psychiatrist on the research team. Participants were shown a total of 40 images (20 neutral, 20 anxiety-inducing; R = 0.5) during the fMRI sessions. There were 4 fMRI runs (Figure 1).

The images were shown in sets (groups of 5 images) as opposed to intermingled in the hopes of producing a more sustained emotional state and allowing for more distinct readings. The ordering of the image sets repeated halfway through (back-to-back of the anxiety-inducing images in the example above) is to control for participants becoming accustomed to image ordering. The images appeared on a projected screen that was reflected into the scanner for participants to view. 0.5% BOLD signal difference between conditions (*p* < 10–6) will be considered a detectable change [effect = 0.005; (30)].

Anatomical reference images were captured with the phase encoding direction collected sagittally from anterior to posterior. These images were captured with T1-weighted high-resolution magnetization prepared rapid acquisition gradient echo (MPRAGE) images with 0.8 × 0.8 × 0.8 mm3 isotropic voxels. A 256 mm field of view (FOV), 2,500 ms repetition time (TR), 2.22 ms echo time (TE), 8-degree flip angle, and a 320 × 320 mm matrix resolution. Following this, T2*-weighted gradient-echo echo-planar imaging (GE-EPI) with 3.0 mm 3.0 × 3.0 × 3.0 mm3 isotropic voxels was used for the stimuli-exposed image acquisitions in an anterior to posterior direction. A 192 mm FOV, 2500 ms TR, 28.4 ms TE, 90-degree flip angle, and a 64 × 64 mm matrix resolution. A multi-band acceleration factor of 2 was employed with 170 volumes being captured. Following the GE-EPI imaging, 2 short spin-echo field map scans were captured from anterior to posterior, then posterior to anterior. These images used a 192 mm FOV, 8000 ms TR, 66.0 ms TE, 90-degree flip angle, 180-degree refocus flip angle and a 64 × 64 mm matrix resolution. All images used a bandwidth of 1,500 Hz.

To un-distort images, the GE-EPI fMRI data was mapped to a non-distorted set of GE images from the same participant. Next, the non-distorted GE images were mapped to the T1-weighted MPRAGE image. Finally, the T1-weighted MPRAGE was mapped to the MNI standardized brain template (31). In doing this, the GE-EPI fMRI data were mapped to the MNI template with maximum accuracy.

### 2.4. Training

The therapists involved in care delivery were all graduate students and research assistants trained in psychotherapy delivery and supervised by a psychiatrist on the research team who has extensive experience in electronically delivered psychotherapy. All therapists were taught the standard care pathway, the aim, and the content of each therapeutic session. Moreover, they were provided sample homework from previous patients and were asked to provide feedback as practice. Feedback templates were developed by the primary therapist and reviewed by a psychiatrist on the research team throughout. This feedback varied between sessions and was personalized for each patient’s homework. Before feedback was submitted to the participant, it was read, edited, and approved by a therapist supervisor on the research team. Training occurred through video calls and exercises with feedback.

### 2.5. Outcomes

The primary outcome measure was changed in neural activation levels between baseline and post-treatment. This was collected through detectable changes in BOLD values from the fMRI scans at baseline and post-treatment (week 16). The secondary outcomes were changes in symptom severity, quality of life, and functioning. Changes in symptom severity were evaluated using clinical symptomatology questionnaires (Y-BOCS; OCI-R; (23), (24)). Changes in quality of life were measured using the Q-LES-Q-SF (32). Changes in levels of functioning will be measured using the SDS (33). All questionnaires will be collected directly through OPTT at baseline, after session 8, and post-treatment (week 16). Additionally, participant perception and experience of the therapy program and the online platform were evaluated.

### 2.6. Compliance

As with all mental health disorders, treatment compliance is always an area of focus when designing interventions. Participants had the importance of treatment compliance explained to them during the informed consent process along with participants needing to submit their homework assignments through OPTT before gaining access to their next treatment session. From a previous meta-analysis conducted, the estimated completion from in-person psychotherapy is approximately 75% (34). Additional meta-analyses found treatment adherence for online psychotherapy to be between 61 and 66% with no significant difference from the in-person psychotherapy (35–39). A study investigating the efficacy of a 10-session e-CBT program for OCD had a mean completion of 7.28 sessions (40). From previous research using OPTT, participants completed over 8 sessions on average, with over half of participants completing all sessions. In a previous project using e-CBT for patients with generalized anxiety disorder, 90% of participants completed 10–12 weeks of the 12-week program, with over 75% of participants being retained for a 12-month follow-up (41).

### 2.7. Analysis

**Figure 2:**
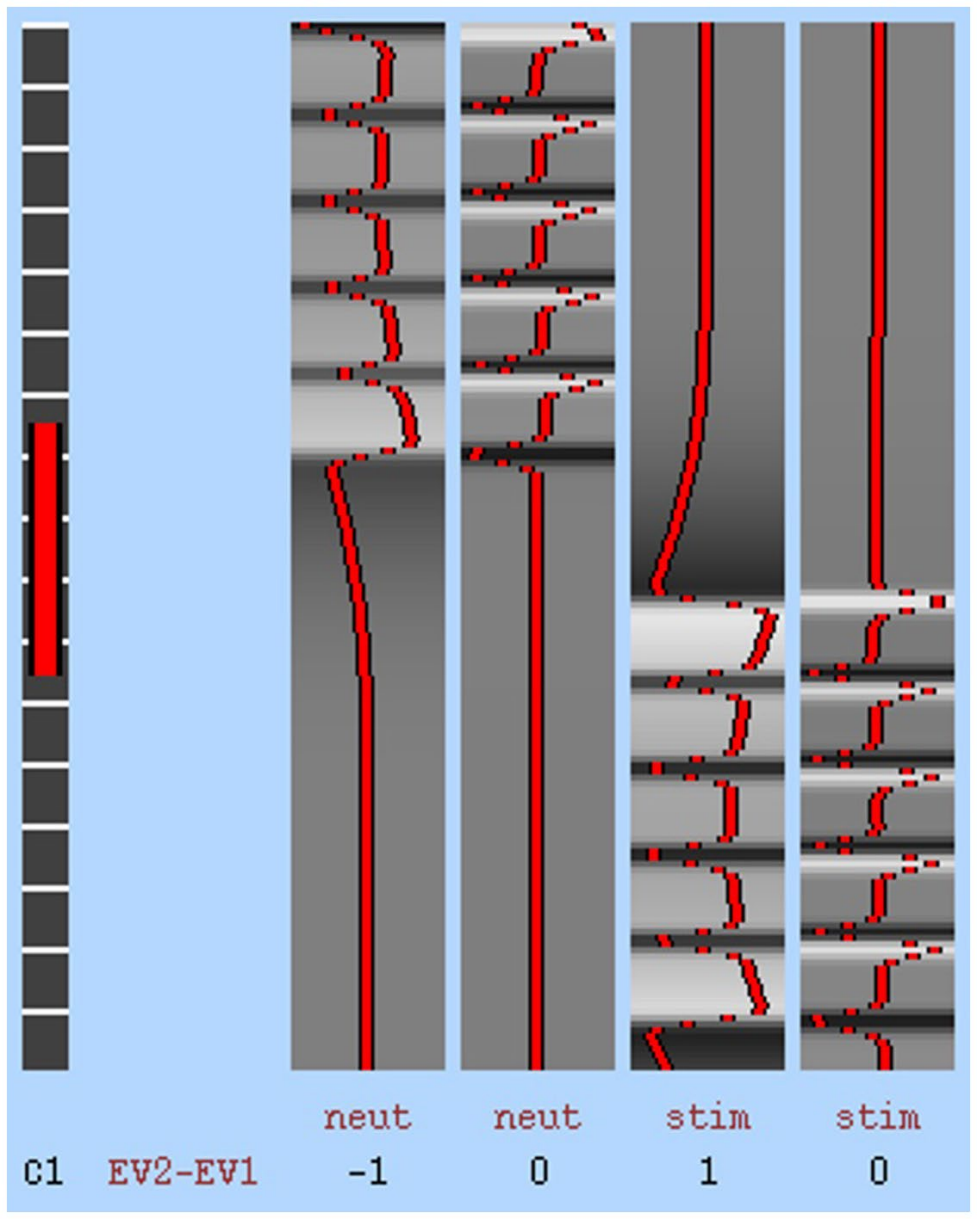
Stimulus onset timing matched the expected hemodynamic response.

DELETED “Neuroimaging analysis was conducted with a whole-brain approach.” All participant raw DICOM images and BOLD data were preprocessed with steps including motion correction, slice-timing correction, smoothing, registration, and normalization, mapped to the MNI T1 2 mm Human Template. Preprocessing For the fMRI data (primary outcome), a 0.5% (effect = 0.005) change in BOLD hemodynamic response function was considered a detectable signal variation between conditions (*p* > 10–6). An estimated paradigm of expected BOLD response was created to calculate the correlation between real signal and expected signal to detect noise using a general linear model (Figure 2; S = βX + e; S = time-series data, β = value for each pattern, X = set of time-series patterns, e = residual). The general linear model provided a β value for each term in the basis set and a T-value for each β. A first-level analysis computing the BOLD contrast between conditions (i.e., neutral and stimulating images) of each run (i.e., 1, 2, 3, 4) was conducted, followed by a higher-level analysis, evaluating the mean BOLD contrast of all runs at each timepoint for each participant. The individual participant means were then concatenated into an overall BOLD contrast over the runs and at both time points. The overall BOLD contrasts were then compared between scanning periods (i.e., baseline, post-treatment) using one-sample paired t-tests, assuming a normal distribution. Mean BOLD values for the left and right sides of each region of interest (ROI; precuneus, cingulate, thalamus, occipital fusiform gyrus, lingual gyrus, and orbitofrontal cortex) were averaged to find a bilateral value. These bilateral values were calculated for each timepoint and condition and then contrasted against each other. Realignment parameter regressors for the testing conditions were implemented (42, 43). Effects at each condition entered a group analysis using a random-effects model (44). Missing data points were accounted for in the analysis with usable questionnaires and fMRI data using the linear model. Preprocessing, first-level analysis, and higher-level analysis were conducted with a combination of packages in Nipype (FSL, AFNI, ANTs, Nilearn) and Brain Voyager (45–50). Motion correction was done through AFNI, slice timing was done through FSL, registration was done through ANTs, and smoothing and filtering were completed through Nilearn. Registration was completed through both FSL and ANTS.

For questionnaire scores (secondary outcomes), mean scores were calculated at each time point (baseline, mid-point, post-treatment) and averaged to find the overall mean, SD, and SE. 2-way paired t-tests were conducted with *p* = 0.05 for each questionnaire with comparisons between baseline and mid-point, mid-point and post-treatment, and baseline and post-treatment to test for significance. Additionally, the means of each time point were used to calculate the effect size between the previously mentioned time points, with Cohen’s d used as the primary effect size, with significance defined as *d* < 0.20 = very small effect, *d* > 0.20 = small effect, *d* > 0.50 = moderate effect, *d* > 0.80 = large effect. Using a Pearson Correlation Coefficient, the correlation between the questionnaire score and the BOLD response was evaluated. Data outliers will be defined as 3.29 SD away from the mean on scores.

Skew and kurtosis were analyzed assuming a normal distribution in the questionnaire and fMRI data at all collection time points. Age and sex variables were considered in knowledge creation and translation. Thematic analysis of participant perception and experience of the online program and platform was used.

### 2.8. Ethics and privacy

The pilot study received approval from the Queen’s University HSREB. Only the care providers involved in the care of the participant had access to their information. Participants were only identifiable by an identification number on the OPTT platform for analysis and hard copies of consent forms with participant identities were stored securely on-site and will be destroyed 5 years after study completion. Only anonymized data was provided to the analysis team members. OPTT is compliant with the Health Insurance Portability and Accountability Act, Personal Information Protection and Electronic Documents Act, and Service Organization Control – 2. Additionally, all servers and databases are hosted in Amazon Web Service Canada cloud infrastructure which is managed by Medstack to assure all provincial and federal privacy and security regulations are met. OPTT only collects anonymized metadata to improve its service quality and provide advanced analytics to the research team. OPTT encrypts all data, and no employee has direct access to patient data. All encrypted backups are kept in the S3 storage dedicated to Queen’s University.

## 3. Results

### 3.1. Participants

Eleven Participants were deemed eligible and enrolled between September 2021 and February 2022, all receiving baseline fMRI scans. Four of these participants did not complete the program, with two of them never beginning treatment. These two patients were removed from the analysis. The other two patients dropped out after completing the fourth week of therapy feeling that the online format was not a right fit for them. This left seven (*n* = 7) patients that completed the 16-week e-psychotherapy program. All but one was able to complete both baseline and post-treatment scans, with one no longer being eligible as they became pregnant. However, this participant was able to complete the post-treatment symptomatology questionnaires, providing data on the efficacy of the program.

At baseline, eight of the nine patients who began treatment were female (89%) with a mean age of 30.78 (SD = 14.18; Table 2). Participants were screened initially by a trained professional to confirm a diagnosis and assess the dominant obsessive thoughts of the patient, which was used to develop the imaging bank for stimulating photos in the fMRI procedure. Patients presented with obsessions including contamination (*n* = 2), negative events to family (*n* = 2), order (*n* = 3), checking (*n* = 4), religion (*n* = 1), general sexual intrusive thoughts (*n* = 1), and child-related sexual intrusive thoughts (*n* = 1). Personalized image banks were constructed accordingly in consultation with a psychiatrist on the research team.

**Table 2:** Behavioral data for each participant across collection time points.

ID	Sex	Age	Status	Y-BOCS			OCI-R			Q-LES-Q-SF			SDS		
				T0	T1	T2	T0	T1	T2	T0	T1	T2	T0	T1	T2
P_1	F	66	Completed	37	28	23	70	70	63	33	40	38	30	30	22
P_2	F	32	Completed	23	25	22	27	28	27	44	34	39	16	16	11
P_3	F	24	Completed	21	21	14	40	39	24	27	38	53	19	13	12
P_4	*Non-Starter*														
P_5	M	27	Completed	15	14	9	17	11	9	40	52	51	16	9	7
P_6	F	28	Dropout W5	18	NA		29	NA		51	NA		10	NA	NA
P_7	F	28	Dropout W5	21	NA		37	NA		23	NA		9	NA	NA
P_8	F	34	Completed	11	8	9	13	8	9	52	54	59	16	9	5
P_9	F	18	Completed	27	27	19	37	37	29	51	48	51	24	23	24
P_10	*Non-Starter*														
P_11	F	20	Completed	14	11	10	23	25	11	37	52	64	15	13	12

T0 = baseline (week 0), T1 = mid-point (week 8), and T2 = post-treatment (week 16).

### 3.2. Behavioral data

**Figure 3:**
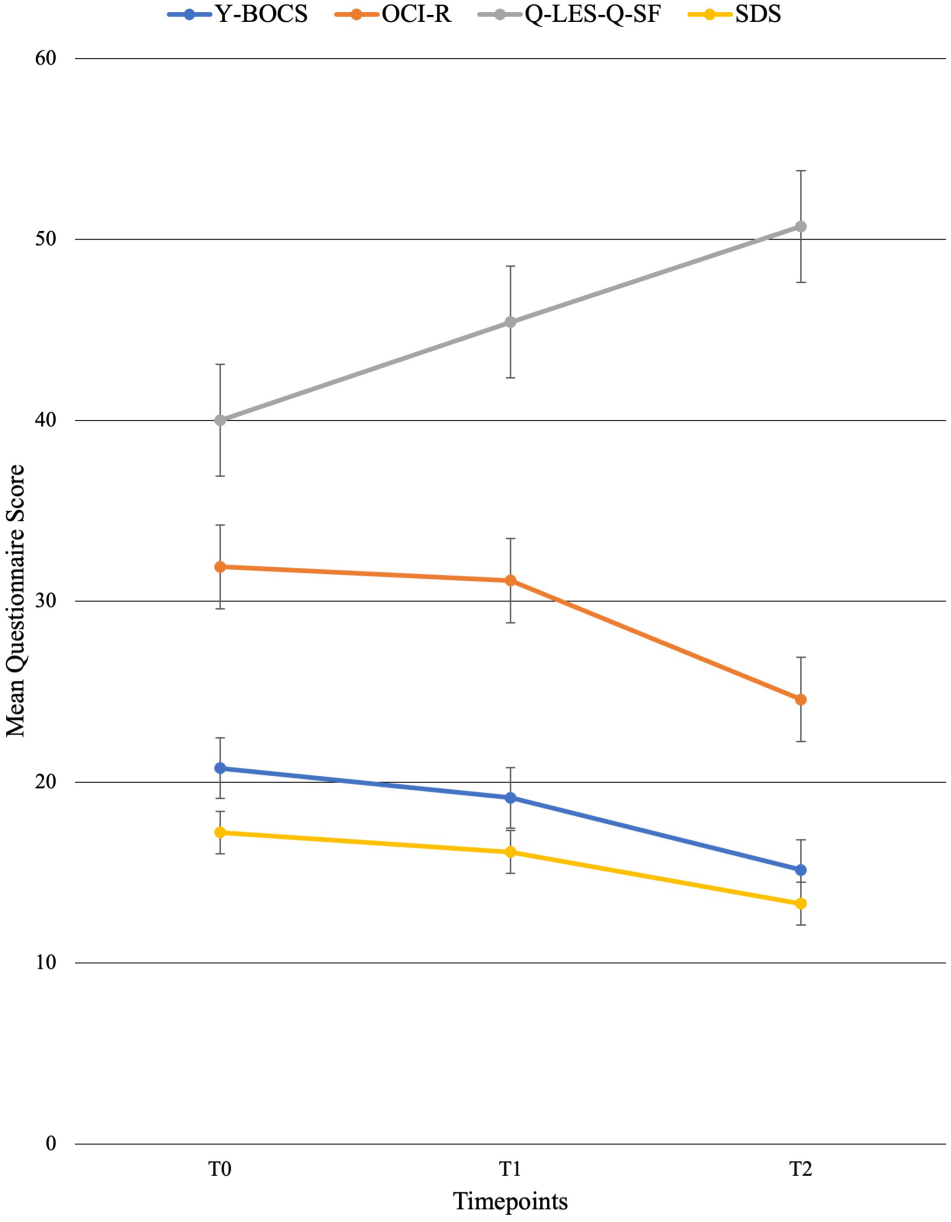
Mean behavioral questionnaire scores across time points with standard error bars. T0 = baseline (week 0), T1 = mid-point (week 8), and T2 = post-treatment (week 16).

For individual participant scores, refer to Table 2. Effect size measurements between baseline and post-treatment can be found in Table 3. Regarding symptom severity, at baseline, participants (*n* = 9) presented with a mean Y-BOCS of 20.78 (SD = 7.82). Midway through treatment (week 8), the mean Y-BOCS score decreased to 19.14 (*n* = 7; SD = 8.11; *t* = 1.36; *p* = 0.22). At post-treatment (week 16), these symptom severity scores significantly decreased from the mid-point; to 15.14 (*n* = 7; SD = 6.15; *t* = −3.29; *p* = 0.02). Overall, the Y-BOCS score decreased by an average of 28.88% through the course of treatment, a significant improvement and large effect size (*t* = −3.54; *p* = 0.01; *d* = 0.80; Figure 3). For OCI-R, patients (*n* = 10) presented with a mean baseline score of 31.9 (SD = 15.97), followed by a mid-point (week 8) score of 31.14 (*n* = 7; SD = 20.80; *t* = −1.12; *p* = 0.31). At post-treatment collection, participants (*n* = 7) reported a significant difference in mean score from the mid-point of 24.57 with a small effect size (SD = 19.03; *t* = −2.76; *p* = 0.03; *d* = 0.42), a 24.63% decrease from baseline (*t* = −4.01; *p* = 0.01; Figure 3).

**Table 3:** Effect size measurements between baseline and post-treatment for behavioral questionnaire scores.

Questionnaire	Cohen’s *d*	Glass’s Δ	Hedges’ *g*
Y-BOCS	0.80	0.72	0.79
OCI-R	0.42	0.46	0.42
Q-LES-Q-SF	1.09	1.06	1.08
SDS	0.57	0.60	0.58

d < 0.20 = very small effect, d > 0.20 = small effect, d > 0.50 = moderate effect, d > 0.80 = large effect.

Regarding changes in quality of life, no significant changes were seen across time points. Participants (*n* = 10) presented with a mean Q-LES-Q-SF score of 40.0 (SD = 10.12). This quality-of-life rating increased to a mean of 45.43 (*n* = 7; SD = 7.98; *t* = 1.42; *p* = 0.20) at mid-point (week 8). At post-treatment, participant (*n* = 7) quality of life further improved from mid-point to a mean score of 50.71 with a large effect size (SD = 9.57; *t* = 2.22; *p* = 0.07; *d* = 1.09), equating to a 25.36% improvement from baseline (*t* = 2.18; *p* = 0.07; Figure 3).

Finally, regarding the level of functioning and disability, participants presented with a mean SDS score of 17.22 (SD = 6.53) at baseline (*n* = 9). At the mid-point (week 8), this means significantly decreased to 16.14 (*n* = 7; SD = 7.76; *t* = −2.67; *p* = 0.04). This was followed by a significant improvement from mid-point to post-treatment (*n* = 7; mean = 13.29; SD = 7.16), a significant 22.86% improvement from baseline with a moderate effect size (*t* = −4.32; *p* < 0.01; *d* = 0.57; Figure 3).

### 3.3. Imaging data

**Figure 4:**
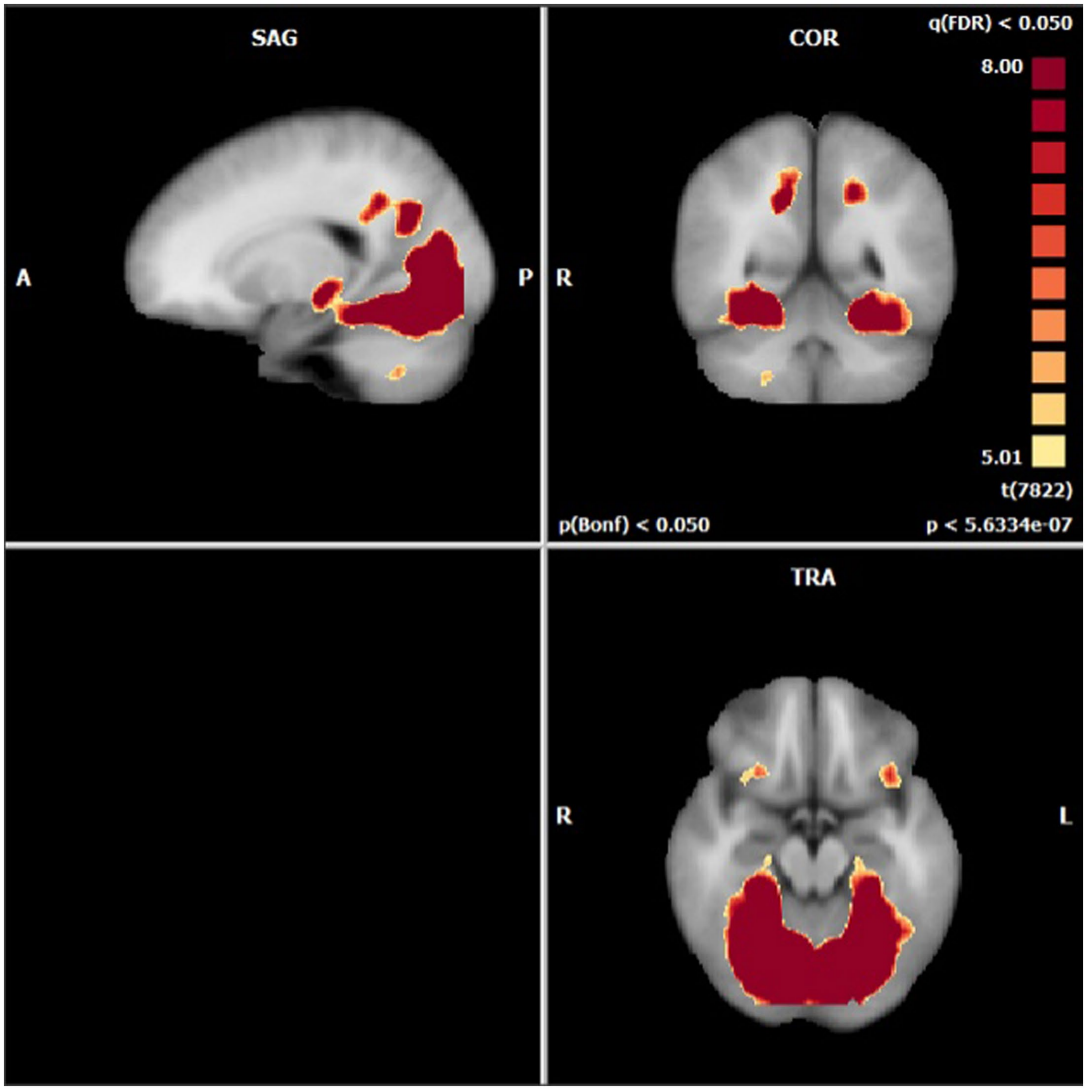
Areas presenting a significant difference in mean BOLD activation when contrasted across neutral and stimulating conditions.

Anatomical imaging and BOLD data were collected for all patients (*n* = 11) at baseline. Following the removal of non-starters (*n* = 2), dropouts (*n* = 2), and those no longer eligible for scans (*n* = 1), post-treatment scans occurred. For analyzing the change in BOLD data across time points, only patients who completed data collection at baseline and post-treatment (*n* = 6) could be included. Across time points, there were no statistically significant differences found in the BOLD contrast between baseline and post-treatment in any cortical regions using a two-way single-sample paired t-test (Table 4). However, as expected, there were significant differences seen between conditions (i.e., neutral and stimulating photos shown during scans). Figure 4 provides a cortical heat map showing the areas of significant differences found in BOLD changes when contrasting mean activation of neutral and anxiety-inducing stimuli through the extracted time course signal of each area (Figure 4).

**Table 4:** 2-way paired *t*-test scores for each bilateral region of interest evaluating changes in BOLD activation during each condition between baseline and post-treatment.

Bilateral Region	Neutral	Stimulating
Precuneus	0.11	0.88
Cingulate	0.96	0.12
Thalamus	0.54	0.99
Occipital Fusiform Gyrus	< 0.01	0.81
Lingual Gyrus	0.11	0.28
Orbitofrontal Cortex	0.62	0.80

While there were no statistically significant differences found in changes between periods, there were interesting changes noted in bilateral activations. In the precuneus, a 39.57 and 11.86% decrease in BOLD activation were noted from baseline to post-treatment for neutral and stimulating conditions, respectively. In the cingulate, a 3.60 and 292.07% increase were noted post-treatment for neutral and stimulating conditions. However, in the cingulate, no activation on the right side was noted, allowing for analysis of the right cingulate only. In the thalamus, a 23.38 and 0.47% decrease occurred after the e-CBT program for the neutral and stimulating conditions, respectively. A large decrease of 178.55% and a 4.72% decrease were found in the occipital fusiform gyrus following treatment for the neutral and stimulating conditions. Within the lingual gyrus, similar decreases of 23.12 and 18.48% were observed for the neutral and stimulating conditions, respectively, following therapy. Finally, in the orbitofrontal cortex, an increase of 21.77% during the neutral condition and a decrease of 14.62% during the stimulating condition were observed at post-treatment scans, compared to baseline mean bilateral BOLD activation values. For a breakdown of changes in BOLD activation between time points and conditions in individual left and right sections of each of the previously mentioned regions, refer to Table 5. For specific mean activation values and cluster sizes at all time points for each condition and each ROI, please refer to Table 6.

**Table 5:** Left and right % changes in mean BOLD activation across time points and conditions for each region of interest.

	*Right side % change*		*Left side % change*	
Region	Neutral	Stimulating	Neutral	Stimulating
Precuneus	−41.24	+13.00	−37.62	−34.93
Cingulate	+3.60	+292.07	*Did not Activate*	
Thalamus	−30.15	+4.21	−15.34	−5.45
Occipital fusiform gyrus	−24.88	+0.13	−17.93	−9.04
Lingual Gyrus	−23.72	−17.96	−22.53	−21.81
OFC	−42.69	−17.55	+131.88	−11.34

**Table 6:** Cluster size and mean BOLD values in each region of interest at each imaging data collection timepoint.

	*Right side mean (SE)*					*Left side mean (SE)*				
Region	Voxels	T1_N	T1_S	T2_N	T2_S	Voxels	T1_N	T1_S	T2_N	T2_S
Precuneus	257	0.57 (0.22)	0.32 (0.28)	0.34 (0.18)	0.36 (0.11)	842	0.49 (0.16)	0.35 (0.19)	0.30 (0.160)	0.24 (0.10)
Cingulate	842	0.27 (0.16)	0.10 (0.17)	0.28 (0.11)	0.38 (0.14)	*Did not Activate*				
Thalamus	902	0.50 (0.16)	0.38 (0.15)	0.35 (0.13)	0.40 (0.12)	925	0.42 (0.18)	0.36 (0.13)	0.36 (0.08)	0.34 (0.12)
Occipital fusiform gyrus	762	0.17 (0.28)	0.99 (0.30)	0.88 (0.22)	0.99 (0.23)	671	1.15 (0.20)	1.11 (0.16)	0.94 (0.13)	1.00 (0.18)
Lingual gyrus	795	1.41 (0.16)	1.28 (0.18)	1.08 (0.15)	1.05 (0.24)	758	1.44 (0.14)	1.41 (0.10)	1.12 (0.10)	1.10 (0.15)
OFC	250	0.28 (0.06)	0.26 (0.09)	0.16 (0.12)	0.21 (0.07)	493	0.17 (0.08)	0.23 (0.09)	0.39 (0.10)	0.20 (0.14)

T1 = baseline (week 0), T2 = post-treatment (week 8); N = natural images, S = stimulating images.

### 3.4. Therapy feedback

In the final session (week 16) of the e-psychotherapy program, the last question asked for feedback on the course, both positive and negative, as well as specific strategies that the patient found helpful. In general, the positive feedback far outweighed the negative feedback. Patients found the layout of the material easy to follow, as well as the ratio of pictures to text practical and made the content less overwhelming. The systematic course-based structure of care was also a point of emphasis in many of the patient’s positive feedback. Moreover, having specific examples and characters in the modules helped patients relate more to the concepts being taught, providing context and real-world applicability. Having the chat box throughout the week with the care provider was also a welcomed feature, allowing questions and concerns to be addressed.

One patient disliked the online format compared to in-person as they felt a disconnect with the therapist, and found it was easier for them to verbally communicate the more debilitating side of their OCD and intrusive thoughts in person. However, they also pointed out the benefit of having access from home during the COVID-19 pandemic and living in a more remote area, making it difficult to access in-person care. Another patient found the online asynchronous format while more convenient also could make it more difficult sometimes to hold themselves accountable. Another found that some of the content was a bit repetitive, although this is done purposefully to reinforce key concepts. In general, patients found the response-prevention, while the most anxiety-inducing aspect of the course, also the most beneficial to them.

## 4. Discussion

### 4.1. Feasibility

This pilot project was conducted to evaluate the feasibility of examining the effects of e-CBT and ERP on cortical activation in OCD patients. Upon completion of this pilot project, the methodology and protocol developed were found to be feasible in a small sample size as a method to evaluate the effects of psychotherapeutic treatment on cortical activation in OCD patients. Using the pilot data collected along with understanding the feasibility of the described protocol, this can be used as a stepping stone for a larger-scaled randomized-controlled trial (RCT) in the future. The electronic delivery method for the psychotherapy program (OPTT) has been proven feasible in previous work (28, 41, 51) and was again found to be easy to implement and use as an online psychotherapy delivery platform. Regarding functional neuroimaging, the block design with the previously described imaging parameters was effective at capturing high-quality imaging with sensitivity to changes in neural activity. The appointments were able to be conducted within a 1-h time slot, allowing for efficient data collection with larger population size in the future. Within this small sample, of the four participants who did not complete, two were deemed non-starters, with the other two completing a month of e-CBT before deciding the online format was not a good fit for them.

### 4.2. Online psychotherapy intervention

The e-CBT and ERP programs were, in general, well-received by patients, shown by overall positive qualitative feedback, as well as clinical symptom severity improvements and levels of functioning upon completion. There is growing evidence supporting the implementation of online psychotherapy interventions for the treatment of various mental health disorders, citing the increased accessibility these remote programs can provide to patients while increasing care capacity. Patients found the module-based format engaging, intuitive, interactive, and easy to follow while visually appealing. Moreover, patients mentioned appreciating the chat feature, giving them the ability to contact their therapist throughout the week with content-related questions, as well as having access to 24/7 technological support provided by OPTT. These modules and feedback templates will continue to be fine-tuned and adapted over time, allowing for more accessible formats and higher quality of care for patients.

### 4.3. Symptom severity, quality of life, and levels of functioning

Regarding symptom severity, both clinically validated questionnaires (Y-BOCS & OCI-R) provided similar results. While neither of these questionnaires indicated statistically significant symptom improvements from baseline (week 0) to mid-point (week 8), they did report statistically significant improvements from mid-point to post-treatment (week 16) and between baseline and post-treatment. This suggests that while there may not be immediate results seen in the online psychotherapy program, at some point after 8 weeks, significant improvements in symptoms are seen. In future work, additional time points for questionnaire completions should be added (i.e., week 4 and week 12) to elucidate a more specific timepoint where symptom improvement is typically seen. Moreover, long-term follow-ups of 6 months and 1 year would be welcomed additions to the collection protocol, providing insight into the possibility of maintaining benefits from program completion.

Interestingly, while there were statistically significant symptom severity improvements, the analysis failed to reveal any statistically significant changes in quality of life (Q-LES-Q-SF) across any time points. It is important to note that while not statistically significant, there was an approximate 25% increase in the questionnaire score. This finding should be considered when treating patients, understanding that a holistic approach should be taken, finding ways to not just improve symptoms, but also understand how we can ensure an improved quality of life, as they should be treated as separate issues.

Level of functioning and disability showed statistically significant improvements across all time points, suggesting that the reduction in symptom severity could be tied to an improvement in functioning. This is a reasonable thought, as reducing the severity of symptoms in a mental health disorder as debilitating as OCD should result in an overall improvement in the patient’s level of functioning.

It is important to recognize that while these changes in symptom severity, quality of life, level of functioning and disability have all been evaluated regarding statistical significance, clinically significant conclusions have not been made. Moreover, the significant findings in this study should be overstated, as the major limitation in the rigor of these results is the small sample size. As a proof of concept and test of feasibility, the methodology for this study was tested and is promising. Particularly with the neuroimaging results, these investigations typically require much larger samples to be able to make more sound conclusions and should be interpreted as a guide for future work rather than conclusive results. With such a small sample, more work should be done in a large-scale RCT aimed at validating the modules used in this pilot project and investigating the clinical significance of the change in symptoms, quality of life, and levels of functioning. Clinical significance tests can provide insight into whether this method of intervention has a practical, real-world effect that can be felt by this population who deserves the highest quality of care.

### 4.4. Changes in cortical activation

As expected with a sample (*n* = 6) of this size available for post-treatment cortical follow-up, the lack of statistically significant findings is expected. However, the trends seen in changes in mean BOLD activation across tasks and time points are in line with previous work. Generally, the front-limbic circuit, and within this, the orbitofrontal cortex, is hyperactive in OCD patients (13). This is the same for panic disorder patients, with one study showing that CBT could normalize these irregular activation patterns (52). In agreement with this, the pilot project showed a 14.62% bilateral decrease in BOLD activation during neural anxiety processing post-CBT program. Other studies using fMRI to assess the effects of treatment on OCD patients have shown decreases in orbitofrontal cortex activation post-treatment (17–21). While this present pilot did not find a correlation between symptom improvement and change in activation, Yang and colleagues found one between Y-BOCS improvement and OFC activation decrease (21). Additional work using similar symptom provocation tasks as the one used in this pilot showed decreases in the orbitofrontal cortex post-treatment (17). Moreover, similar studies with an HC comparator showed that CBT resulted in the orbitofrontal cortex having a more similar activation to the HC (19, 20, 23).

Within the thalamus, the present study found decreases of 23.38 and 0.48% in BOLD activation during neutral and stimulating conditions post-treatment. The thalamus has been cited as being hyperactive at rest in OCD patients (13), with other symptom provocation studies finding decreased activation post-CBT intervention (17). The precuneus has also been shown to be hyperactive in OCD patients, with studies citing decreases following treatment with psychotherapy in resting-state and symptom provocation fMRI tasks (12, 17, 19, 22). Similarly, decreases were observed in the occipital fusiform gyrus in this pilot, similar to this previous work (12, 17, 19, 22, 23). In the cingulate, the findings from this study, increases in neutral and anxiety-processing activation post-treatment are in agreeance with some work (19, 23) but non-conforming to other studies (17). In the lingual gyrus, this pilot found decreases in both neutral and anxiety-inducing conditions, contrary to previous work (12, 21). While the study was limited in findings of statistical significance, the changes observed, while small, are generally in line with previous work from in-person CBT interventions for OCD patients.

## 5. Conclusion

The present pilot project provides insight into the feasibility and effectiveness of a protocol that uses functional neuroimaging to evaluate the effects of an online psychotherapy program on neural anxiety processing in OCD patients. The protocol provides a proof of concept for a large-scale RCT delivered in the future. From qualitative patient feedback, the online psychotherapy program is intuitive, well-designed, and engaging from a patient perspective, and provides improved efficiency and access to care. Significant improvements in symptom severity and levels of functioning and disability were seen following the completion of treatment, suggesting this is a promising solution to meet the increasing demand for accessible and timely high-quality mental health care. The clinical significance of the online modules and intervention format should be validated in the future through a clinical trial.

While the findings did not provide statistical significance, they were in agreeance with much of the literature surrounding the effects of psychotherapy on cortical activation in OCD patients. To the author’s knowledge, this is the first study to implement an online psychotherapy program to observe the effects of treatment with fMRI on OCD patients. With preliminary findings suggesting similar results to in-person psychotherapy programs, a larger-scale trial could offer more insight into whether this online intervention offers a comparable impact on cortical activation to traditional treatments.

This pilot project sets the stage for a clinical trial further investigating the pathology of OCD through a treatment-centered lens with functional neuroimaging. By understanding the mechanisms of action associated with online psychotherapeutic interventions, innovative treatment plans with high-quality care and improved outcomes can be developed for OCD patients in need of cutting-edge solutions to this debilitating mental health disorder.

## Data availability statement

The raw data supporting the conclusions of this article will be made available by the authors, without undue reservation.

## Ethics statement

The studies involving human participants were reviewed and approved by Queen’s University Health Sciences and Affiliated Teaching Hospitals Research Ethics Board. The patients/participants provided their written informed consent to participate in this study.

## Author contributions

CS conducted the research described in this manuscript under the co-supervision of NA and RM, conducted all data collection, was responsible for the cognitive behavioral therapy delivery and patient feedback, under the supervision of NA, and additionally, he was responsible for conducting analysis and writing the manuscript of this thesis. NA and RM provided guidance and expertise on the development of the trial and methods, data collection and analysis, and composition of the manuscript. NM was responsible for the development of the electronically delivered cognitive behavioral therapy modules used in this treatment program. JN assisted in the analysis of functional magnetic resonance imaging data. TS and DC assisted in protocol development and data analysis. NA is a co-founder of the care delivery platform used in this study (OPTT) and has shares in the company. All authors contributed to the article and approved the submitted version.

## Funding

CS is a recipient of the Canada Graduate Scholarship – Masters (CGS-M) from the Canadian Institutes for Health Research.

## Conflict of interest

The authors declare that the research was conducted in the absence of any commercial or financial relationships that could be construed as a potential conflict of interest.

## Publisher’s note

All claims expressed in this article are solely those of the authors and do not necessarily represent those of their affiliated organizations, or those of the publisher, the editors and the reviewers. Any product that may be evaluated in this article, or claim that may be made by its manufacturer, is not guaranteed or endorsed by the publisher.
